# Effects and safety of herbal medicines on patients with overactive bladder: A protocol for a systematic view and meta-analysis

**DOI:** 10.1097/MD.0000000000017005

**Published:** 2019-09-13

**Authors:** Jin Zhou, Chenglong Jiang, Peng Wang, Shen He, Zirong Qi, Shujun Shao, Yinshan Tang

**Affiliations:** a Beijing University of Chinese Medicine Shenzhen Hospital(Longgang), Shenzhen; b Foshan Hospital of Traditional Chinese Medicine; c The Integrated Chinese and Western Medicine Hospital of Guangdong Province, Foshan; d School of Acupuncture-Moxibustion and Tuina, Beijing University of Chinese Medicine, Beijing; e Department of Rehabilitation in Traditional Chinese Medicine, The Second Affiliated Hospital of Zhejiang University School of Medicine, Hangzhou, China.

**Keywords:** herbal medicines, overactive bladder, protocol, systematic review

## Abstract

**Background:**

Overactive bladder (OAB) is a common disease in the urinary system. The morbidity is increasing annually. Herbal medicines have been pervasively used in the therapy of OAB. However, systematic review or meta-analysis has not been found to assess the effects and safety of herbal medicines in curing OAB at present. Hence, the systematic review is conducted to scientifically and methodically evaluate the value of its effectiveness and safety of herbal medicines on OAB.

**Methods:**

We will collect all randomized controlled trials (RCTs) to assess the effectiveness and safety of herbal medicines on OAB. The RCTs will be searched from the electronic databases, including 7 English databases, consisting of PubMed, Excerpta Medica Database (EMBASE), MEDLINE, Web of Science, Cochrane Library, SpringerLink, and WHO International Clinical Trials Registry Platform (ICTRP), and 4 Chinese databases, namely Wanfang Chinese database, China National Knowledge Infrastructure (CNKI), Chinese Biomedical Literature Database (CBM), and Chinese Scientific Journal Database (VIP), others valid search strategy will be adopted. All the RCTs will be assessed from the databases establishment to July 2019. We will perform the meta-analysis of RCTs when the dissertation is appropriate. We will conduct an assessment including data synthesis, bias risk, and subgroup analysis by means of Review Manager software (RevMan) V.5.3.5 software while the setting condition is met.

**Results:**

This systematic view and meta-analysis will generate a summary based on the current relevant research to calculate the effects and safety of herbal medicines in promoting the therapy of OAB. Furthermore, it will provide a high-quality synthesis for participants who use herbal medicine to cure OAB.

**Conclusion:**

The summary of our systematic view will verify whether herbal medicines intervention could be an effective and safety approach in curing OAB.

## 1 Introduction

Overactive bladder (OAB) has the clinical characteristic urinary symptoms of urgency, frequency, accompanied with nocturia, with or without urge incontinence, excluding proven infection and other obvious pathologic, according to the International Continence Society (ICS) in 2010 and the American Urological Association (AUA) guidelines.^[1,2]^ Urodynamic testing presents the overactive detrusor or the urethrovesical dysfunction. According to the clinical symptoms, OAB is generally divided into 2 categories, OAB dry and OAB wet.^[3]^ The character of OAB dry is 4 episodes of urgency at least in the past 4 weeks, accompanying with frequency 8 more times every day or using 1 coping behaviors to control bladder function. On the basis of OAB dry, OAB wet accompany with ≥3 episodes of urinary incontinence in the previous 4 weeks that have nothing with stress incontinence clearly.^[3]^

OAB was listed as one of the top 10 chronic diseases in the United States (US), with a higher incidence than other common chronic disease. Given the rebarbative symptom, it has caused a terrible impact on OAB patient, s quality of daily life. Multiple studies have shown that OAB could induce higher morbidity risk of depression and diabetes, urinary tract infection, hypertension, and osteoporosis, accompanying with the poorer quality of sleep, less social interactions, and lower work productivity.^[4–6]^ As the newest report, the prevalence rate of OAB was 16.9% in the National Overactive Bladder Evaluation (NOBLE) research and 30% in OAB on Physical and Occupational Limitations (OAB-POLL) in women aged ≥16 years in US.^[7]^ In Asia, the prevalence of OAB is 22.1% in women and 19.5% in men.^[4]^ The EPIC study, that is the largest population-based survey to assess prevalence rates of OAB, provided an OAB prevalence rate in adults aged ≥40 years of 17.4% for women in Europe, with tending to increase with age.^[8,9]^

The total cost in US of OAB was estimated proximately $65.9 billion, of which $14.6 billion was spent on losing productivity due to OAB, which results in vast increasing financial burden on OAB-related medical care system.^[10,11]^ According to newly administrative database analysis, the healthcare costs for OAB patients treated with anticholinergic therapy were estimated to be 33% higher than non-OAB patients, leading to an extra consumption of $1746 per person each year on average.^[12]^

Based on above mentioned points, we draw a conclusion that OAB has caused a vast financial burden on medical system and society. If we can seek an effective means to treat OAB, it will greatly lessen society burden.

The scientists have not found the nature pathophysiology of OAB currently, there are several hypotheses based on detrusor overactivity that have been accepted widely as follows:

(1) Myogenic hypothesis: urgency originating from the detrusor;
(2) Urotheliogenic hypothesis: urgency originating from the bladder urothelium/suburothelium;
(3) Urethrogenic hypothesis: urgency stemming from the urethra;
(4) Supraspinal hypothesis: urgency originating from the brain and brainstem;
(5) Detrusor underactivity.^[13]^

Thus, it needs further research to find the definite pathogenesis of OAB, contributing to the preventment and treatment of the disease.

The American Urological Association (AUA) recommends a 3-line treatment for OAB in 2015, the first-line treatments is behavioral therapies (Grade C), such as pelvic floor muscle training as well as bladder training, reducing the intake of caffeine and fluid, weight-reducing, but it is too time-consuming and difficult for patients to insist the therapy.^[2]^ Additionally, less than half participants following it reach satisfactory effect.^[14]^ Second-line treatments refer to pharmacologic management, including anti-muscarinics and β3-adrenoceptor agonists (GradeB). Third-line treatments is invasive neural regulation. However, the use of it requires various consideration and discussion.

As we known, the oral anti-muscarinics is a therapy that OAB patients prefer to choose. The adverse drug events (AEs) in antimuscarinic involves widely, the details are as follows: blurry vision, dry mouth, and constipation as well as hypertension in beta-3 agonists.^[15]^ These AEs cause patients cannot stick to the therapy, which generate a relapse of OAB and lower the quality of life (QOL).^[16]^

In the AUA/SUFU Guideline Amendment emphasized that the treatment goals of OAB is to maximize manage symptoms and improve patients QOL, meanwhile minimizing adverse events and easing patients burden. Based on current studies, herbal medicines can alleviate symptoms of OAB greatly, avoiding side effects.^[2,17–20]^

Although plenty of clinical trials about herbal medicines intervention to OAB, there still lack of systematic evaluation and meta-analysis about it, s efficacy. The aim of the research is to comprehensively evaluate the effects and safety of herbal medicine^,^s intervention in clinical research, furthermore offering the field to advance medical treatment to OAB.

## 2 Methods

This registration number is CRD42019129583. The protocol report has been registered on the international prospective register of systematic review (PROSPERO). We will conduct the protocol rigidly under the guidelines of Preferred Reporting Items for Systematic Reviews and Meta-Analyses protocols (PRISMA-P).^[21]^

### 2.1 Inclusion and exclusion criteria for study selection

#### 2.1.1 Types of studies

Only the RCTs of herbal medicine treating OAB will be included in this study, without placing the constraint on publication status and writing language. The studies which compare or analyze the effectiveness among different components of herbal medicine will be excluded. Studies without sufficient information about the randomized method or process, the animal mechanism studies, qualitative studies, uncontrolled trials and reviews and case reports will be excluded.

#### 2.1.2 Types of participants

Participants who are diagnosed with OAB will be enrolled, regardless of age, gender, race, and education background (not considered). However, participants accompany with infection and other pathologic change will be excluded.

#### 2.1.3 Types of interventions

*Experimental interventions:* We will include all type of herbal medicine (there is no restriction of dosage, frequency, administration method, or duration of treatment) or herbal medicine combine other treatment to assess the effects of herbal medicine on OAB patients.

*Control interventions:* The control group will include treatments such as conventional care (drugs, exercise, education, behavioral approach, etc), placebo or blank control group. Additionally, studies compare herbal combine with the other treatment (excluding herbal medicine) will also meet the control group including criteria.

#### 2.1.4 Types of outcome measures

*Primary outcomes:* The frequency of daytime urination and nocturnal urination, urgency and urge incontinence, mean urine volume average 24 hours on the basis of the 3-day bladder diary that participants should accomplish it before and after the treatment period (week 6) will be adopted as the primary outcomes.

*Secondary outcomes:* The scores of overactive bladder symptom score (OABSS) and QOL questionnaire, and Maximum cystometric capacity (ml).

### 2.2 Search methods for the identification of studies

#### 2.2.1 Electronic searches

We will screen the comprehensive literature from relevant electronic databases, including 7 English databases, consisting of PubMed, Excerpta Medica Database (EMBASE), MEDLINE, Web of Science, Cochrane Library, SpringerLink, and WHO International Clinical Trials Registry Platform (ICTRP), and 4 Chinese databases, namely Wanfang China database, China National Knowledge Infrastructure (CNKI), Chinese Biomedical Literature Database (CBM), and Chinese Scientific Journal Database (VIP). All the RCTs will be assessed from the database establishment to March 2019. The search items include overactive bladder, OAB, Bladder overactive, Overactive bladder syndrome, Urge inconvenience, overactive detrusor, overactive, Detrusor overactivity, Bladder instability, Detrusor instability, Muscarinic Antagonists, herbal, drug therapy, herbal treatment, traditional treatment, used alone or in combination to ensure the same searching terms in both Chinese and English database, an equivalent translation of the search terms will be adopted. The detailed selection process for PubMed is presented in Table 1 and we will make relative modifications in accordance to the requirements. Beyond that, for the full article is unavailable, we will try our best to resort to the authors for it.

**Table 1:**
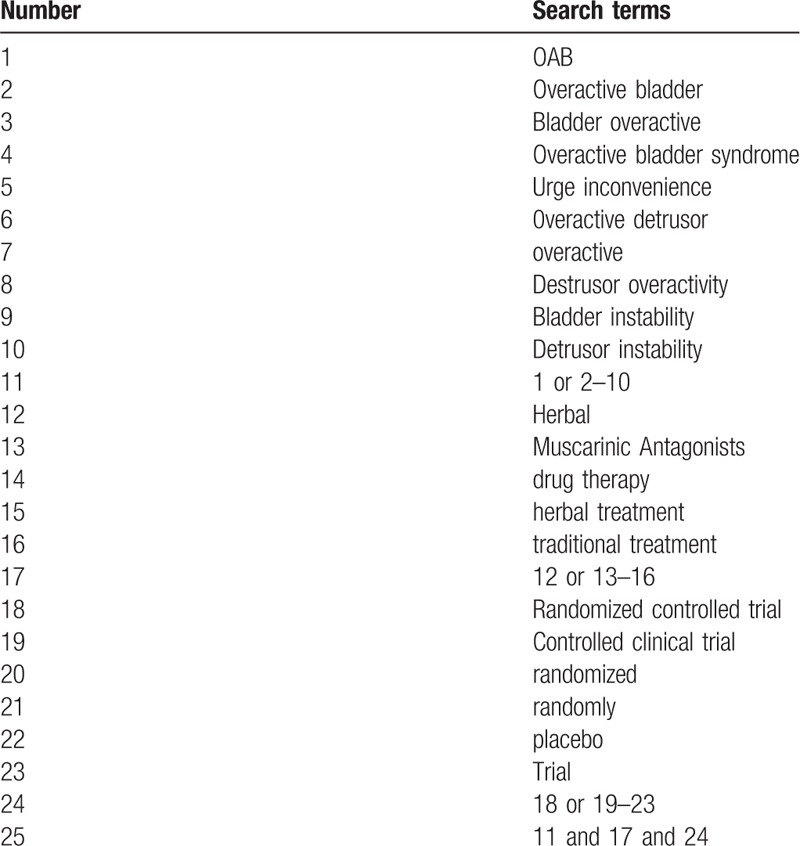
Search strategy for PubMed.

#### 2.2.2 Searching other resources

Additionally, related conference papers, or dissertations also will be manually retrieved as complement. Meanwhile, the study captain of relevant unpublished research will be contacted. At the same time, we will also search the WHO ICTRP to pick out new study relevant to the theme.

### 2.3 Data collection and analysis

#### 2.3.1 Study selection

To ensure that all reviewers can conduct the study precisely, we will organize a session for reviewers, the teacher who has been trained and gained certifications in Chinese Cochrane Centre will teach them the approach of it and fully comprehend relative information of the study for insight on the purpose and process of the study.

**Figure 1:**
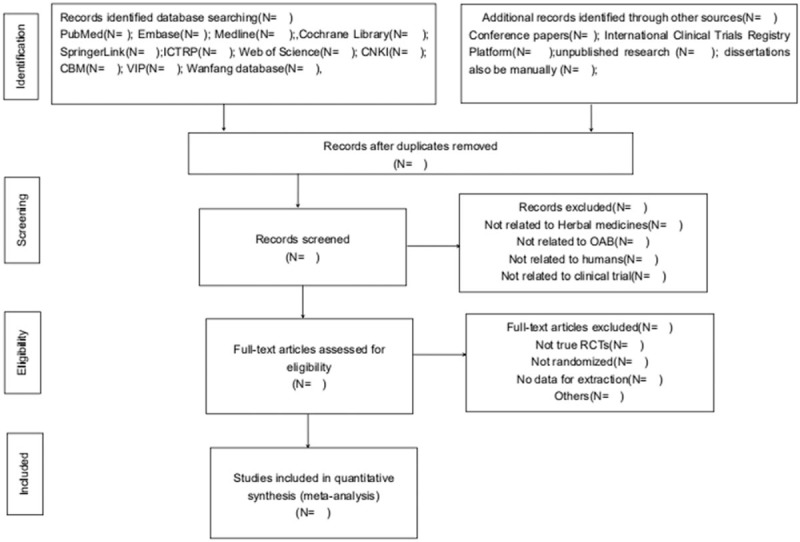
Preferred Reporting Items for Systematic Reviews and Meta-Analysis (PRISMA) flow diagram.

Two independent researchers (Jin Zhou and Chenglong Jiang) will investigate all potential relevant literatures and screen from title to abstracts respectively to extract eligible articles, eliminating repeated 1. Subsequently, reviewing the full-text and comprehensively considering to determine eligible studies. The all studies that reviewers chosen will be discussed in the group until the final team consensus reached. The selection process is tabulated in the following PRISMA flow diagram (Fig. 1)

#### 2.3.2 Data extraction and management

Taking advantage of electronic form, the extract data from studies will be recorded by Jin Zhou and Chenglong Jiang independently, according to predefined criteria. The content should consist of these items: the first and corresponding author, publication time, trial designate, the type of herbal medicine intervention, control intervention, characteristics of participants, duration of follow-up, outcomes, adverse effects, and other specific information. Disagreements will be settled after group discussion and if necessary, consulting experts and arbiter. We will appeal to the corresponding authors for future details if necessary.

#### 2.3.3 Assessment of bias risk and quality of included studies

The assessment of potential bias risk and quality of included literature will be respectively executed by two reviewers (Jin Zhou and Chenglong Jiang) based on the guidelines of Cochrane collaboration^,^s tool. Six characteristics will be evaluated: random sequence, generation, allocation concealment, the blinding method for patients, researchers and outcomes assessors, incomplete result data addressed, and selective outcome reporting and other issues. This the bias risk will be classified into 3 types, low, high, and unclear bias.

#### 2.3.4 Measurements of treatment effect

With respect to continuous data, the mean difference (MD) will be adopted to analysis of clinical efficacy, the relative risk (RR) will be used to evaluate the treatment effect for categorical data. The standardized MD with 95% confidence interval (CI) or RR with 95% CI will be employed to express the effect values.

#### 2.3.5 Managing missing data

If the missing or incomplete data are detected involved in trials, the 2 reviewers (Jin Zhou and Chenglong JIANG) will get in touch with the corresponding author via email or phone to integrate the data. If it does not succeed, we will conduct our analysis based on the current data. Meanwhile, we will organize a group meeting to discuss the impact of the missing data and present the conclusion in the result section.

#### 2.3.6 Assessment of heterogeneity

Based on the guidelines of the Cochrane Handbook for Systematic Reviews of Interventions, we will adopt a standardized chi-squared test (α = 0.1) and *I*^2^ value to assess the heterogeneity. If I^2^ ≤ 50%, indicating that there is no statistical heterogeneity, and a fixed-effect model can be chosen. While, if *I*^2^ > 50%, presenting the study exist substantial heterogeneity, and a random-effect model can be employed.

#### 2.3.7 Assessment of reporting biases

When the number of trials included in the study is more than 9, a visual asymmetry on the funnel plot will be performed to determine whether there exists a reporting bias.

#### 2.3.8 Data synthesis

We will adopt Review Manager software (RevMan) V.5.3.5 to synthesize and analyze the available date, provided by Cochrane Collaboration. If *I*^2^ < 50%, we will choose a fix-effect model to evaluate MD and RR. If *I*^2^ ≥ 50%, we regard there have plentiful heterogeneity, the random effect model will be chosen to synthesize the data. Both of them will adopt 95% confidence intervals (CIs). If obvious heterogeneity is found between studies, the subgroup analysis will be performed to explore the possible reasons contributing to the statistical heterogeneity. Additionally, descriptive analysis will be presented.

#### 2.3.9 Subgroup analysis

When there is a substantial study, subgroup analysis will be conducted to detect the heterogeneity among groups. We will take sex and age of patients, different forms of herbal medicine and control interventions, and course of treatment, the severity of OAB into account.

#### 2.3.10 Sensitivity analysis

We will conduct a sensitivity analysis to identify whether the results are robust in the review based on following aspects: sample size, missing data, and methodologically quality, getting rid of the low-quality studies. The sensitivity analysis will exclude studies which have nonrandom generation to improve the conclusion.

#### 2.3.11 Grading the quality of evidence

Under the guidelines of Grading of Recommendations Assessment, Development and Evaluation (GRADE) guidelines, the quality of evidence for primary outcomes will be assessed. The classification of evidence will be graded into very low, low, moderate, or high 4 levels.

#### 2.3.12 Dissemination and ethics

Not involving in individual or private message, the systematic has no necessary to acquire the ethical approval and informed consent, we will share our study of our review for peer-reviewed journals and present it at conferences or relevant meeting for the clinician.

## 3 Discussion

The symptoms of OAB is urgency, frequency, accompanied with nocturia, with or without urge incontinence, which have affected the patient's daily life seriously. Currently, the government has invested substantial cost into the therapy of OAB, and the cost increasing annually, which hinder the social development. Unfortunately, the key problem is scientists have not found a satisfying approach to prevent and cure OAB up to now.

Herbal medicines root in China, has been extensively used in clinic for ages. According to Traditional Chinese Medicine (TCM) theory, the pathogeny of OAB is widely considered to be the deficiency of kidney qi and damp-heat pouring downward, qi is viewed to have the function of containment, meaning the ability to control the liquid substances. We take advantage of the therapy: tonify kidney and qi or heat-cleaning and dampness-eliminating to treat OAB depend on diagnosis and treatment, which is the superiority and soul of TCM. Also, herbal medicines can strengthen the condition of the viscera, qi, blood, Yin and Yang in the whole body to promote health and keep it in a balance state. Additionally, as a treatment to OAB, herbal medicine therapy has gained increasing popularity across the globe in recent years. A mass of studies have testified that herbal medicines is effective and safety for alleviating the symptoms of OAB; however, there is no article to evaluate the efficacy and safety of herbal medicines in treating OAB so far.^[16–19]^ We provide a protocol of a systematic review to comprehensively analyze the effectiveness and safety of herbal medicines for OAB on evidence-based literature, both RCTs from home and abroad will be collected, with the purpose of establishing a more convincing preference for clinicians treating OAB with herbal medicines.

There might have some limitations. First, the inclusion of literatures only adopts Chinese and English medical databases. Therefore, we may slip some relevant literatures published in other languages. Second, sex and age of patients, different forms of herbal medicine and control interventions, and course of treatment, the severity of OAB and study quality may give rise to high statistical heterogeneity.

## Author contributions

Jin Zhou and Chenglong JIANG contributed to the conception of the research. Jin Zhou and Yinshan Tang designed the search strategy and drafted the manuscript ultimately. Jin Zhou and Chenglong JIANG will search all relevant research then select eligible studies independently. Peng WANG, Shen HE, Zirong QI will evaluate the bias risk. Shujun SHAO is responsible for managing missing data. Yinshan Tang is the arbitrator of this study, in charge of any disagreement and ensure that the review progress smoothly. All the authors participated in this protocol carefully revised the final manuscript before submission, and confirmed on the publication of it.
